# *In silico* modelling of neuron signal impact of cytokine storm-induced demyelination

**DOI:** 10.1098/rsob.240138

**Published:** 2024-09-04

**Authors:** Geoflly L. Adonias, Harun Siljak, Sasitharan Balasubramaniam, Michael Taynnan Barros

**Affiliations:** ^1^ Walton Institute for Information and Communication Systems Science, South East Technology University, Waterford, Ireland; ^2^ Department of Electronic and Electrical Engineering, Trinity College Dublin, Dublin, Ireland; ^3^ School of Computing, University of Nebraska-Lincoln, Lincoln, NE, USA; ^4^ School of Computer Science and Electronic Engineering, University of Essex, Colchester, UK

**Keywords:** neuron, action potential, demyelination, molecular communications, cytokine storm, Hodgkin–Huxley

## Abstract

In this study, we develop an *in silico* model of a neuron’s behaviour under demyelination caused by a cytokine storm to investigate the effects of viral infections in the brain. We use a comprehensive model to measure how cytokine-induced demyelination affects the propagation of action potential (AP) signals within a neuron. We analysed the effects of neuron-neuron communications by applying information and communication theory at different levels of demyelination. Our simulations demonstrate that virus-induced degeneration can play a role in the signal power and spiking rate, which compromise the propagation and processing of information between neurons. We propose a transfer function to model the weakening effects on the AP. Our results show that demyelination induced by a cytokine storm not only degrades the signal but also impairs its propagation within the axon. Our proposed *in silico* model can analyse virus-induced neurodegeneration and enhance our understanding of virus-induced demyelination.

## 1. Introduction

The recent outbreak of the coronavirus disease 2019 (COVID-19) pandemic caused by the severe acute respiratory syndrome coronavirus 2 (SARS-CoV-2) has shaken society as a whole, leaving a long-lasting impact on people’s health. As a result, scientists from multidisciplinary fields have been joining efforts and resources towards the study of not only the epidemiological characteristics and transmission dynamics of the virus, but also of the physiological damage to the human body as the infection is prolonged. SARS-CoV-2 is well known for affecting primarily the respiratory system, and it can potentially leave lifelong sequelae in tissues and organs. Furthermore, there has been an increase in works that suggest SARS-CoV-2 may be able to invade the nervous system [1–4] and elicit neurodegeneration [5,6] where sequelae may have other detrimental effects on patients’ lives post-infection.

Viruses that present the ability to infect nerve cells are known to exhibit neurotropic properties and can also be called neuroinvasive. By infecting cells in the nervous system and replicating themselves within it, these viruses can negatively impact neurological functions and even cause severe nerve damage by triggering a pro-inflammatory immune response [6]. Unfortunately, SARS-CoV-2 is not the only virus that exhibits this kind of behaviour; for example, it has been shown that the Zika virus (ZKV) [7] can infect the peripheral nervous system (PNS) and sometimes spread to the central nervous system (CNS). Furthermore, viruses such as the human immunodeficiency virus (HIV) [8] can infect the CNS and cause neuroinflammation, induced by an immune response of the body, consequently leading to neurodegeneration [9]. A growing amount of data from the past few years support the hypothesis that chronic damage caused by different infectious agents can lead to neurodegeneration [10], as early experimental models of virus-induced demyelination have indicated [11].

**Figure 1.**
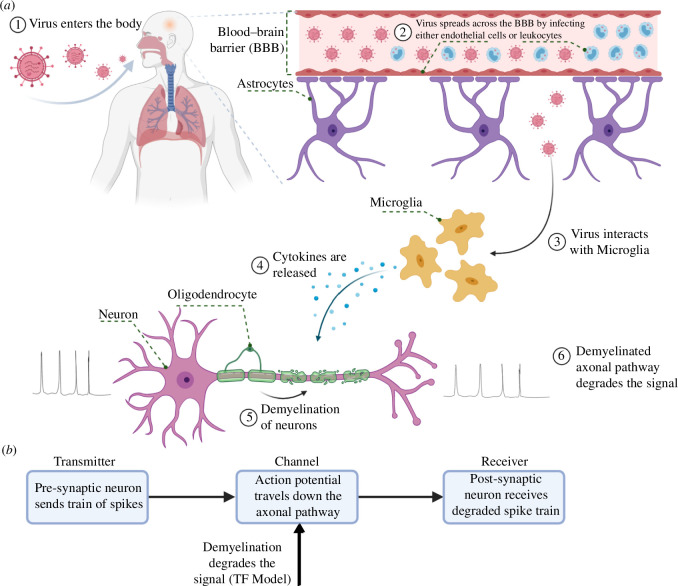
Pathogenesis of a virus-induced demyelination. (*a*) Depiction of the viral entry into the respiratory system, passage through the BBB and effects of demyelination to the neurons. (*b*) The system model captured by our model in this paper.

Viruses are known for causing dramatic structural and biochemical changes to the host cell, by hijacking and exhausting its machinery for replication until, eventually, the cell is killed. This viral manipulation of a host cell provokes neuroinflammatory defence mechanisms that can be characterized by numerous toxic-metabolic derangements, such as cytokine storms (figure 1*a*). Such inflammation could potentially lead to several types of neurodegeneration, including demyelination. As cytokines are released to fight the infection, healthy tissues could be affected as a ‘collateral damage’ of the fight against infectious agents [12]. Other types of coronavirus have been known to cause demyelination, such as the murine coronavirus (M-CoV), which has been identified to cause demyelinating disease and, even after the virus is cleared from the CNS, the demyelination can continue for a few months [12]. This behaviour also matches findings on SARS-CoV-1, which reports a decrease in viral titers as clinical disease worsens [13]. M-CoV is a type of coronavirus of the same genus (*Betacoronavirus*) as SARS-CoV-2, and it is believed to be 43.8–48% similar to this novel coronavirus [14]. SARS-CoV-2, when compared to SARS-CoV-1, triggers lower levels of interferons and pro-inflammatory cytokines and chemokines. However, it is capable of infecting and replicating a significantly higher amount of viruses in human tissues [15].

This article presents a systems theory-based analysis of the demyelination (either directly or indirectly) caused by viral infections from the perspective of molecular communication (MC), and more specifically a neuro-spike MC. The goals of this article are: (i) to propose a model extension coupled with computational analysis on the Hodgkin–Huxley (HH) formalism to account for the effects of cytokine storms indirectly caused by viral infections; (ii) to provide insights on how the demyelination will affect the neuronal information along the axonal pathway; and (iii) to provide a transfer function (TF) model that describes the effects of the membrane action potential (AP) caused by the degeneration of the myelin sheath. This TF can be considered fundamentally as a model of the demyelination process itself. It is intrinsically tied with the behaviour of equivalent resistance–capacitance (RC) circuits, but at the same time has a reduced complexity as it opts for exponential asymptotics. Given that complex cascades of equivalent simple blocks (in our scenario, myelin sheaths) are traditionally well approximated by first-order TFs with time delay and the underlying physical rationale, our model joins the family of established, applicable biophysical TF models. We expect that these analyses not only could pave the way for more in-depth studies that can support *in vitro* and *in vivo* experimental works on the neurological effects induced by neurotropic viral infections but also serve as a prediction tool that can help to guide future experimental works. It may even be necessary that, as the demyelinating scenarios get more and more biologically plausible, future works may have to fine-tune some of the parameters (e.g. regarding the type of cell or the intensity of cytokine storm-induced demyelination) of the proposed model in order to have as accurate an analysis as possible.

The contributions of this work are as follows:

— *A mathematical model that serves as a building block for a TF of the signal propagation*. We present a model inspired by the well-documented fact that viral infections trigger cytokine storms and lead us towards a general TF that accounts for the propagation of the signal. The cytokine storms are counter-measures of the immune system against the infection. We investigate the signal power, the signal attenuation and magnitude-squared coherence (MSC) analysis of the axonal pathway as a communication channel.
— *An analysis of the effects of demyelination on the neuronal AP propagation*. We conduct a variety of analyses, such as latency, attenuation and spiking rate, on the spike trains that pass through a demyelinated pathway. Furthermore, we also analyse how the intensity of cytokines storms correlates with the amount of attenuation present in the signal at the output of the neuron.
— *A TF model that accounts for the effects of the demyelination-induced attenuation*. We propose a transfer function that describes the transition of a healthy to an unhealthy neuron. The model accounts for sheath-by-sheath demyelination which affects membrane potential, peak times and spike width. This should lead to more in-depth analysis and open a new view on demyelination modelling, especially those triggered by a neurotropic viral infection and how it affects the transmembrane molecular exchange (i.e. exchange of ions and release of neurotransmitters) of the neurons.

## 2. Background and literature review

The virus replication process exhausts host cells leading to the activation of the immune system, which calls for the work of macrophages. Those are types of cells of the immune system, and their resident in the brain is the microglia [1,4]. Macrophages secret pro-inflammatory cytokines such as interleukin-1 (IL-1), interleukin-6 (IL-6) and tumour necrosis factor-alpha (TNF- α ) aiming to fight the infection. These cytokines exert cytotoxic effects on neurons and glial cells (e.g. oligodendrocytes), damaging the myelin sheaths which are responsible for providing support and insulation to axons [16]. The dynamics of cytokine storms and their efficiency in fighting infections without further cellular damage become of increased attention to measure the neuroinflammatory effects of COVID-19. Ludwig *et al*. [17] found that as the neuropathy becomes more severe, the serum concentration of TNF- α and IL-6 becomes higher. This matches more recent results found by Chen *et al*. [18] and Song *et al*. [19] with respect to cytokine levels concerning the severity of COVID-19 cases. They identified a positive correlation between the levels of cytokines and the severity of the COVID-19 cases, meaning that the more severe the COVID-19 cases were, the higher were the levels of cytokines, which can lead to demyelinating lesions [5,20].

On the other hand, the dynamics of a cytokine storm can be similar for different inflammatory scenarios, yielding the need for a more general approach that evaluates cytokine dynamics. An example is the work of Waito *et al*. [21] in which they match recordings of 13 different cytokines with a model of non-linear ordinary differential equations. Likewise, Yiu *et al*. [22] analysed the dynamics of cytokine storms and provided evidence for how cytokines induce or inhibit other cytokines. Additionally, some pieces of literature report the effects of cytokine storms on specific neuronal and non-neuronal structures. For instance, the work of Bitsch *et al*. [23] shows a negatively correlated relationship between the amount of microglia-produced TNF- α and the concentration of myelin oligodendrocyte glycoprotein (MOG). It shows that the more TNF- α there is, the less MOG oligodendrocytes will produce, compromising the myelin sheath structure. On the other hand, Redford *et al*. [24] showed how the number of axons found in sciatic nerves is affected. They found that the more the concentration of TNF- α is increased, the more axons are found to be damaged. However, the complete biophysical models of these various effects to neurons caused by infection, particularly infections from COVID-19, require urgent attention since correct treatment procedures for acute infection damage can benefit from mathematical modelling.

In the past decade, MC has been improving biological models by accounting for the communication of cells using their signalling mechanisms as information carriers [25–27]. MC is a new field that is looking to characterize and engineer biological cells using concepts from communication engineering and networking [28–30], it bridges electrical and communications engineering, molecular biology and biomedical engineering, and provides complete end–end models of biophysical transmission of molecules, their propagation, and their reception [31–35]. A recent survey [36] reports numerous works concerning the use of MC for the analysis and modelling of infectious diseases. However, accounts for the effects of infections are still missing from a biophysical approach even within MC models, as the COVID-19 effects are many, and the molecular interactions with the body can be used to predict the behaviour of a population of cells, even tissues and organs. The emergence of novel MC models for biophysical processes, such as the demyelination induced by COVID-19, delivers an in-depth analysis of tissue behaviour that is needed for treatments based on synthetic biology. Even further targeted drug delivery technology can alleviate the cytokine storms effects on neurons and provoke the restoration of the myelin sheath [37].

## 3. End-to-end computational model for a cytokine storm-induced demyelination

Models that describe the dynamics and evolution of cytokine concentrations, without regard to the cells that secrete or are affected by them, have been proposed by Yiu *et al*. [22]. This model was extended by coupling together with a neuronal model that implements the behaviour of a myelinated axon [38]. By extending these models, we performed simulations with the default parameters of the cell (such as the length and diameter of each of their compartments) based on the original model. Therefore, the myelin sheath properties concerning their morphological characteristics were not modified or changed. We studied and quantified the propagation of APs on a Layer 5 (L5) pyramidal neuron mimicking damage to its myelin sheath. All simulations were performed with extensions to the NEURON Simulator [39].

### 3.1. Cytokine signalling in microglia

The growth and decay of an individual cytokine’s response to its given initial state are first represented by a second-order, linear, time-invariant ordinary differential equation. Denoting the serum concentration ρ(t) in pg ml^−1^, and its rate of change $\Delta_{\rho }(t)$ , it can be represented in a vector-matrix form as follows:


(3.1) $$\begin{array}{l} \left[\begin{array}{c} \dot{\rho }(t)\\ \dot{\Delta_{\rho }}(t) \end{array}\right]=\left[\begin{array}{cc} 0 & 1\\ -a & -b \end{array}\right]\;\left[\begin{array}{c} \rho (t)\\ \Delta_{\rho }(t) \end{array}\right],\;\left[\begin{array}{c} \rho (0)\\ \Delta_{\rho }(0) \end{array}\right]\text{given,} \end{array}$$

where the initial concentration, ρ(0)=0 , is referenced to the cytokine’s basal level, and the initial rate of change, $\Delta_{\rho }(0)$ , is stimulated by the TGN1412 infusion (refer Yiu *et al.* [22] for further details). Also, a and b are positive constants that express the sensitivity of the cytokine’s acceleration to concentration and rate of change.

The cytokine’s response modes are characterized by the eigenvalues, $\lambda_{1}$ and $\lambda_{2}$ (rad day^−1^), of the stability matrix of the system, and this is as follows:


(3.2) $$\begin{array}{l} \left[\begin{array}{c} \dot{\rho }(t)\\ \dot{\Delta_{\rho }}(t) \end{array}\right]=\left[\begin{array}{cc} 0 & 1\\ -\lambda_{1}\lambda_{2} & \left(\lambda_{1}+\lambda_{2}\right) \end{array}\right]\;\left[\begin{array}{c} \rho (t)\\ \Delta_{\rho }(t) \end{array}\right],\;\left[\begin{array}{c} \rho (0)\\ \Delta_{\rho }(0) \end{array}\right]\text{given.} \end{array}$$

The parameters are chosen to minimize the error between the cytokine concentration and the clinical trial measurements performed by Yiu *et al.* [22]. For TNF- α , $\lambda_{1}=\lambda_{2}=-2.63$ and $\Delta_{\rho }(0)=32821$ . In this work, we will be investigating the inflammatory effects of TNF- α , as this cytokine is well-known for its pro-inflammatory properties.

### 3.2. Conduction through a myelinated axon

**Figure 2.**
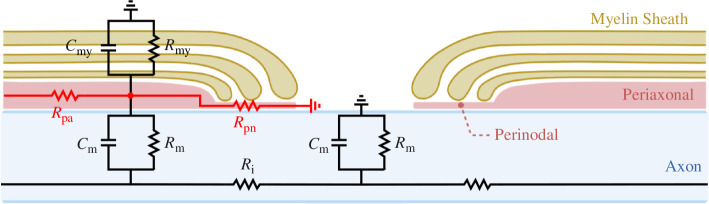
Schematic of the equivalent myelinated axon circuit, with added periaxonal ( $P_{a}$ ) and paranodal ( $P_{n}$ ) axial resistances [38].

It is well known that some neurons contain a myelin sheath wrapped around sections of their axons. Myelin sheath helps propagate electrical impulses, known as APs or spikes, and avoid significant attenuation due to parallel synaptic processes. According to Cohen *et al*. [38], the myelin sheath dynamics can be described using circuit theory (figure 2). This circuit is then coupled with the HH circuit model [40], which describes the membrane potential dynamics in neurons.

Axial resistance, $R_{i}$ ( $\Omega \cdot {\text{cm}}^{-1}$ ), of the axon core, can be defined as the ratio between axial resistivity, $r_{i}$ ( Ω⋅cm ), and the cross-sectional area of the axon core, and this is described as


(3.3) $$R_{i}=\frac{4r_{i}}{\pi d^{2}},$$

where d (nm) is the axon core diameter. Let $\delta_{pa}$ (nm) be the radius of the periaxonal space, then


(3.4) $$\delta_{pa}=\frac{1}{2}\left[-d+\sqrt{d^{2}+\left(\frac{4r_{pa}}{\pi R_{pa}}\right)}\;\right],$$

where the axial resistance in the periaxonal space, $R_{pa}$ (G $\Omega \cdot {\text{cm}}^{-1}$ ), is calculated as the ratio between periaxonal resistivity, $r_{pa}$ ( Ω⋅cm ), and periaxonal cross-sectional area. In this case, the axon core cylinder is surrounded by both the periaxonal and perinodal spaces forming a ‘larger’ axon cylinder of diameter $d+2\delta_{pa}$ , thus


(3.5) $$R_{pa}=\frac{r_{pa}}{\pi \delta_{pa}(d+\delta_{pa})},$$

and an analogous calculation can be performed for $\delta_{pn}$ (nm), $r_{pn}$ ( Ω⋅cm ) and $R_{pn}$ (T $\Omega \cdot {\text{cm}}^{-1}$ ).

Recognizing that a myelin sheath is an in-series compaction of n layers, the radial resistance of the sheath, $R_{my}$ (k $\Omega \cdot {\text{cm}}^{2}$ ) is the sum of the resistance of each myelin membrane, $R_{mm}$ (k $\Omega \cdot {\text{cm}}^{2}$ ), formulated as


(3.6) $$R_{my}=\sum_{i=1}^{n}R_{mm_{i}},$$

and the radial capacitance of the myelin sheath, $C_{my}$ ( $\mu \text{F}\cdot {\text{cm}}^{-2}$ ), may vary inversely to the sum of the capacitances of each of its composing membranes, $C_{mm}$ ( $\mu \text{F}\cdot {\text{cm}}^{-2}$ ). Thus,


(3.7) $$\frac{1}{C_{my}}=\sum_{i=1}^{n}\frac{1}{C_{mm_{i}}},$$

where the resistance and capacitance of a single myelin membrane are $R_{mm}$ and $C_{mm}$ , respectively. Based on this, equations (3.6) and (3.7) can be represented in terms of the number of myelin lamellae, $n_{my}$ , as follows:


(3.8) $$n_{my}=\frac{R_{my}}{2R_{mm}}=\frac{C_{mm}}{2C_{my}}.$$

For a more detailed analysis of the myelin sheath’s modelling, the reader is referred to the work of Cohen *et al*. [38]. Each of the lamellae that compose the whole myelin sheath is modelled with the same set of parameters. This is basically an assumption that each myelin lamellae is identical to the other and that the degeneration caused by the demyelination affects the myelin sheath proportionally.

### 3.3. Cytokine-induced demyelination

As previously discussed in figure 1, the literature indicates that, as microglia cells release pro- and anti-inflammatory cytokines to fight infection, demyelination may also occur as a side effect and, consequently, compromise the neuronal signal propagation. There are numerous pieces of evidence linking cytokine storms to neurodegeneration [17,21–23]; however, to the best of our knowledge, none of them goes as far as linking the storm’s intensity to an approximate number of myelin lamellae, $n_{my}$ . A linear regression ( R=0.508 , p=0.001 ) applied by Ludwig *et al*. [17] to their own data reveals a proportional relationship between the severity of the neuropathy, ζ (in our scenario, the neuropathy is the demyelination) and the serum concentration of TNF- α , ρ(t) , as


(3.9) ζ(t)=20.727−0.9228⋅ρ(t).

Even though the cytokine dynamics by Yiu *et al.* [22] are triggered by a specific antibody, the proportionality presented in equation (3.9) is also suggested by the works of Hartung [41] and Empl *et al*. [42]. In this relationship, the stronger the cytokine storm is, the more severe the degeneration of the myelin sheaths. Furthermore, in this work, we are using data from Cohen *et al.* [38] as a reference to indicate healthy myelin with 13 lamellae. In the study by Ludwig *et al.* [17], the severity of the neuropathy was defined by scoring four nerve functions as ‘ 0 ’ for typical values of the nerve, ‘ 1 ’ for affected nerves (either decreased amplitude and/or decreased nerve conduction velocity) and ‘ 2 ’ for no stimulation possible. As we are looking at it from the perspective of $n_{my}$ , we had to re-scale these scores by applying linear interpolation to indicate approximately how many sheaths are being degraded. Therefore, building on top of their findings we hypothesize that the worst-case scenario would have a score of 8 (four nerve functions multiplied by two—no stimulation possible) which indicates $n_{my}=0$ and the best-case scenario would have a score of 0 indicating $n_{my}=13$ . Similar experiments conducted by the scientific community potentially on top of this work can re-scale ζ(t) depending on their reference of normal myelination as the number of myelin sheaths may vary among different types of neurons from different parts of either the CNS or the PNS.

The model proposed by Ludwig *et al.* [17] addresses neuropathies in the PNS in which peripheral neurons are myelinated by Schwann cells. On the other hand, this work examines neuropathies in the CNS where myelination is mainly controlled by oligodendrocytes. Even though oligodendrocytes and Schwann cells can express different types of myelin proteins, a major difference would be that oligodendrocytes can myelinate several axons at the same time while Schwann cells can wrap around only a single axon at a time [43,44]. In terms of myelination processes, both types of cells are quite similar and the model itself already leaves room for improvement as the parameters may be finely tuned as soon as new evidence is presented in the literature.

### 3.4. A linear model of demyelination

The linearization process aims to find a linear approximation of a nonlinear system (i.e. the HH model) at an equilibrium point. This means that for small variations around said point, the linear system should behave similarly to the nonlinear system [45]. The linear model is the result of the process of linearization applied to the conventional (non-linear) HH model. In this process, the dynamics of the ionic channels are ‘lost’, the components that represent each channel (e.g. variable conductance and a voltage source) are ‘reduced’ to a static conductance and an inductance. As the demyelination only affects the RC circuits coupled to the internodal compartments, the electronic elements for the axon should remain in their default parameters and the myelin sheath RC circuits be affected by the inflammatory effects of a cytokine storm. Based on a given healthy neuron, we want to predict the signal effects on spike trains that are expected from a demyelinated neuron. Let n , the number of the myelin sheaths, be a tuneable parameter of the model (in this section, we refer to $n_{my}$ as n for clarity of index notation). This mechanism is depicted in the block diagram in figure 1*b*.

In telecommunication systems, the study of damage and degradation often asks for a model of damage effects which could explain how healthy (i.e. signals characteristic to the system without damage) transition into faulty signals (i.e. signals characteristic to the damaged system). Observing faulty signals resulting from the propagation of standard signals through a TF superimposed on the original system is a well-established concept; examples of its application include modelling of cables [46]. It makes intuitive sense to observe such a TF as taking the healthy system’s output and delivering the faulty, unhealthy signal as the faulty output, which results in signal degradation for a worsening channel it traverses. In our case, this means that we will construct a TF which, for an input that represents a healthy neuronal signal, delivers an output equivalent to that of a demyelinated neuron. The opposite process, in which an ‘adaptor’ TF would convert a signal from a deteriorated neuron into one of a healthy neuron, is anti-causal as it would have to introduce negative time delays in the signal.

**Figure 3.**
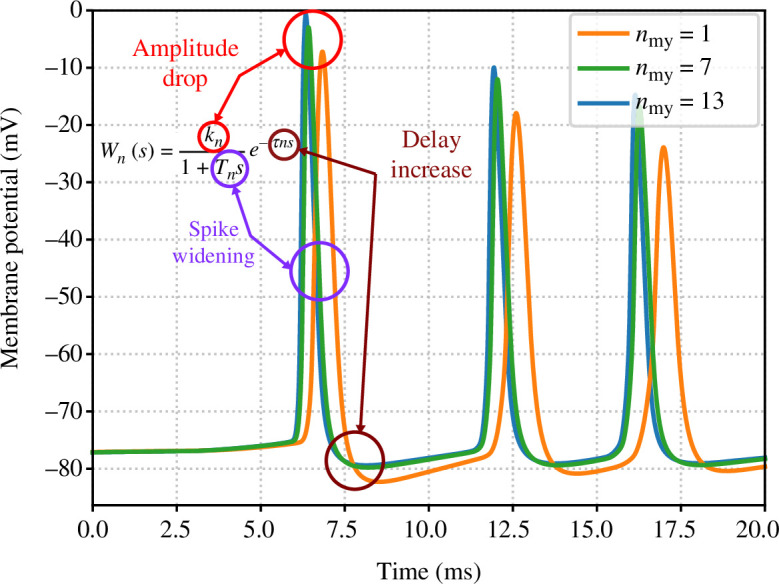
Mapping of the model components and the effects on the propagated signal, and illustration of signal behaviour as it transitions from a healthy state to an unhealthy state.


In this regard, when we speak of our *model*, we have the TF in mind. Our model’s *input* is the series of spikes produced by a healthy pre-synaptic neuron and the model’s *output* is the series of spikes a demyelinated neuron would fire, as illustrated in figure 1*b*. The model *parameters* are factors in the TF; their value depends on the number of myelin sheaths we desire the demyelinated neuron to have. Hence, the number of myelin sheaths is a tuneable scalar value characterizing the model. Now that we have established the processing chain, the choice for the TF is made by observing the general trends in the output signals for various values of n . Namely, as shown in figure 3, the TF emulating the effect of myelin deficiency needs to allow for the attenuation of the signal, widening of the spikes and overall propagation delay. This delay is caused by the demyelination process and the proposed TF does not account for random delays [47]. An obvious candidate is the traditional first order plus time delay (FOPTD) function [48], given by

(3.10) $$W_{n}(s)=\frac{k_{n}}{1+T_{n}s}e^{-\tau_{n}s},$$

where we assume that the parameters $k_{n}$ , $T_{n}$ and $\tau_{n}$ have different values for different values of n . While those values could be tabulated and looked up for specific values of n , we set a more ambitious goal of determining the laws according to which they change. Here, we make a hypothesis that they follow exponential law (namely, $\log\;k_{n}=a_{0}\cdot a_{r}^{n}$ , $T_{n}=T_{o}\cdot T_{r}^{n}$ and $\tau_{n}=\tau_{0}\cdot \tau_{r}^{n}$ ), based on the following reasoning.

Turning a healthy signal into a deteriorated one in our model is a process of cancelling the effect of passing through some of the sheaths. For example, if the healthy baseline signal is achieved with n=13 , emulation of n=10 can be interpreted as undoing the effect of Δn=3 sheaths (i.e. the effect of a cascade of three blocks representing a ‘sheath undo function’). This approximation is good for larger Δn , as it allows for the approximations like $(1+T\bar{s}{)}^{\Delta n}\approx 1+T^{\Delta n}s$ , i.e. giving rise to a $T_{n}∼e^{n}$ law. In §4, we revisit this hypothesis, both in terms of the exponential law’s existence and in terms of the domain of accuracy. Introducing exponential laws for the coefficients in this TF gives us the final form of our TF, which is represented as


(3.11) $$W_{n}(s)=\frac{e^{a_{0}\cdot a_{r}^{n}}}{1+T_{0}\cdot T_{r}^{n}s}e^{-\tau_{0}\cdot \tau_{r}^{n}s}=\frac{e^{a_{0}\cdot a_{r}^{n}-\tau_{0}\cdot \tau_{r}^{n}s}}{1+T_{0}\cdot T_{r}^{n}s}.$$

The final form of equation (3.11) suggests the rationale behind suggesting exponential behaviour of a=log⁡k instead of k itself: in the s -domain, we obtain a function of the form $\frac{e^{\left(\alpha +\beta s\right)}}{\gamma +\delta s}$ , which in turn in the Fourier domain corresponds to $\frac{e^{\left(\alpha +j\beta \omega \right)}}{\gamma +j\delta \omega }$ . Knowing that the behaviour of the myelin circuit originates from connections of R (purely passive, real impedance) and C (purely active, imaginary impedance), it is expected to observe this symmetric real-imaginary coupling of terms.

The knowledge about the equivalent model of the myelin circuit, a ladder of resistors and capacitors supports the choice of FOPTD, and this is known as a convenient model for large RC circuits [49]. Namely, the circuit consists of linear components and as such can be represented by a linear model. Furthermore, with the increasing order of such a linear model, there is a necessity to replace it with a simpler first-order model such as FOPTD as it grows and will keep the complexity of the model low while retaining accuracy.

FOPTD is not an uncommon choice in biophysics, where it has been used to model glucose control [50], because they are simple for identification [48] and for quick and accurate tuning of the controllers that can regulate their behaviour [51]. Nonetheless, our model may help in designing chemical control loops for myelin reinforcement in a similar manner.

To verify the quality of the model, we introduce a metric based on root mean square error (RMSE):


(3.12) $$M_{n}=20\;\log_{10}\left(\frac{{RMSE}_{N,n}}{{RMSE}_{W,n}}\right).$$

Here, ${\text{𝑅𝑀𝑆𝐸}}_{W,n}$ stands for the RMSE of the output of our TF $W_{n}$ compared to the actual output signal for n sheaths if m is the number of samples, which is represented as


(3.13) $${RMSE}_{W,n}=\sqrt{\frac{1}{m}\sum_{i\geq 1}(x_{n,i}-x_{W,i}{)}^{2}}.$$

Analogously, we can find the value for ${\text{𝑅𝑀𝑆𝐸}}_{N,n}$ which corresponds to the RMSE of the output signal produced by another model N compared to the n th actual output signal. The quantity $M_{n}$ is positive where our model $W_{n}$ is more accurate (has lower RMSE) than the model N we are comparing to it.

Certainly, the parameters might eventually change; however, qualitatively speaking, changes in axon structure from different neurons should exhibit similar behaviour to each other. For example, some delay on signal propagation may be introduced due to different axonal lengths [52], but it should not affect the shape of the APs as it is the case with axons that have been demyelinated. Furthermore, the TF works on sub-threshold neuronal signalling and this stimulation technique usually does not lead to the firing of APs even though it can still fire depending on the intensity of the stimuli. Our aim is to apply a similar approach to Khodaei & Pierobon [53] to study the impact on the signalling caused by the demyelination that may have been indirectly caused a viral infection.

### 3.5. Signal analysis

Visually, we first noticed subtle shifts in amplitude (peak potential reached by the membrane) and in time (spikes were taking longer to reach their peak values). We then decided to quantify these shifts, both in amplitude and in time, on average, by proposing a metric that we called 'relative mean shift'. For the analysis on time shift, we consider the points in time where each spike peaked at the input as $T_{in}^{k}$ , where k={1,2,3,...,K} identifies the order of each spike and, $T_{out}^{k}$ as the peak times at the output. Thus, we define the relative mean time shift, $\bar{\delta_{t}}$ (ms), as


(3.14) $$\bar{\delta_{t}}=\frac{1}{K}\sum_{k=1}^{K}(T_{out}^{k}-T_{in}^{k}).$$

Analogously, we can define the relative mean amplitude shift, $\bar{\delta_{v}}$ (mV), with $V_{in}^{k}$ and $V_{out}^{k}$ as the peak amplitudes of spike k at the input (spike peak observed in the soma) and output (spike peak observed in the axon), respectively.

As there is an average shift in time inside the channel itself due to demyelination, we also expect an increase in latency as we decrease $n_{my}$ . In order words, latency is the time interval between the input and the output, and it often occurs due to the channel or network’s intrinsic characteristics. In our scenario, we are looking for the time the first spike peaked at the output, concerning the point when this same spike peaked at the soma of the neuron. Later, analogous to the relationship between $\bar{\delta_{t}}$ and the latency, we decided to look into a potential attenuation on the power of the signal, P (mW), as we have already discussed a relatively heavy mean attenuation in the membrane potential, $\bar{\delta_{v}}$ . Let us define P as


(3.15) $$P=\lim_{J\to \infty }\left(\frac{1}{2J+1}\sum_{j=-J}^{J}|x[j]{|}^{2}\right),$$

where in a set of J samples, x[j] corresponds to the potential of the membrane at the *j*th sample.

We also quantified the attenuation of the signal, A in dB/ 100 m, which is the gradual loss of power of a signal over its propagation through the channel. Depending on the attenuation coefficient, one can calculate more accurately the attenuation in a specific material. For our analysis, we used the generic form of attenuation for RF cables. This decision is based on the fact that the axonal pathway is modelled using cable theory as a leaky cable. Thus,


(3.16) $$A=10\cdot \log_{10}\left(\frac{P_{i}}{P_{o}}\right),$$

where $P_{i}$ and $P_{o}$ , in W , are the input and output power of the signal. In this analysis, the input is the spike train through a healthy neuron and the output would be the spike train through a demyelinated one.

Finally, we also analysed the relation between the reference spike train ( $n_{my}=13$ ) and all other demyelinating scenarios ( $1\leq n_{my}\leq 12$ ). The intention is to understand how much power is being transferred between each pair of signals. With that in mind, we applied a coherence ( $C_{xy}$ ) metric, which is described as


(3.17) $$C_{xy}(\omega )=\frac{S_{xy}(\omega {)}^{2}}{S_{xx}(\omega )S_{yy}(\omega )},$$

where $S_{xy}(\omega )$ is the cross-spectral density between the two signals, and $S_{xx}(\omega )$ and $S_{yy}(\omega )$ are the power spectrum densities of input and output, respectively.

## 4. Computational results and discussion

Let us consider the proportionality between the severity of the neuropathy, ζ(t) , and the number of myelin lamellae, $n_{my}$ , as described in §3.3. Our analysis consisted of an evaluation of the neuronal behaviour and spike propagation under normal circumstances ( $n_{my}=13$ ), followed by an analysis on the demyelination by decreasing $n_{my}$ . The objective is to understand what happens to the neuronal information when travelling through a demyelinated axonal pathway. In other words, we are looking at the signalling within a single neuron that has been impacted by the demyelination on its own, rather than signals that have changed from pre-synaptic neurons that may be affected by cytokine storms. The neuron receives an external current of 3 nA for 15 ms starting at time t=2.5 ms in a 20 ms simulation. The spikes evoked under normal circumstances are considered our input of the channel, and the spikes at the far end of the axon are the output. The axon itself is our communication channel, while the demyelination is acting as an attenuator for the channel, as illustrated in figure 1.

### 4.1. Analysis of a demyelination-induced channel attenuation

**Figure 4.**
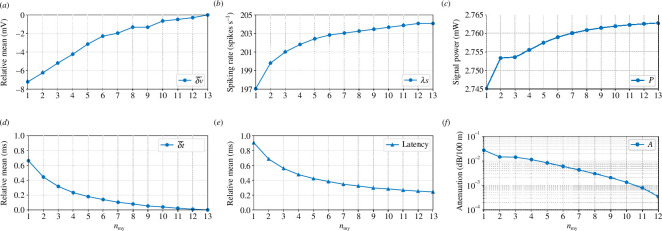
Measurements of attenuation and delay. All results except for (*e*) use the signal $n_{my}=13$ as their input and $1\leq n_{my}\leq 12$ as the output; (*e*) uses the recordings from the soma of the neuron and is compared with $1\leq n_{my}\leq 13$ . (*a*) Relative mean amplitude shifts, (*b*) spiking rate, (*c*) signal power, (*d*) relative mean time shift, (*e*) relative mean latency and (*f*) attenuation.

The results for $\bar{\delta_{v}}$ and $\bar{\delta_{t}}$ from equation (3.14) are shown in figure 4*a*,*d*, respectively. The former shows that membrane potential is on average affected by an eightfold decrease. This matches fundamental computational neuroscience theory on neuronal modelling which states that a neuron gradually leaks a small amount of the input signal as it travels through it, and this ‘leak’ worsens on unmyelinated cables [54,55]. On the other hand, from the latency data (figure 4*e*), we note that there is a subtle ‘lag’ for the signal to travel across the axon even under normal circumstances matching findings in the literature [56] for models of CNS demyelination. This is most likely due to a maximum conduction speed inherent in the axonal membrane itself. As we go from our worst scenario towards a regular healthy myelin sheath, there is a massive decrease of about 73% in the latency.

Figure 4*b* depicts how the spiking rate is affected by the demyelination. As we expected, as the spikes start to get wider and further from each other, the spiking rate gets lower. This corroborates findings on demyelination-induced effects on spiking rate [57]. The rate at which a neuron fires APs is significant for modulating and encoding neuronal information in cognitive, sensory and motor functions.

Figure 4*c* shows that the decrease in signal power is quite subtle, and from the worst-case scenario to the best, there is a difference of less than 20μW . This indicates how demyelination affects the energy consumption per unit time used to propagate the axon’s APs. As the signal starts to get degraded, it is less and less likely a spike would be evoked at the post-synaptic neurons connected to a demyelinated cell [55]. Demyelination does not affect only the post-synaptic neuron by reducing the chances of evoking post-synaptic potential, but it can compromise the spiking rate of the demyelinated neuron itself. Furthermore, we decided to investigate the attenuation caused by the demyelination from a more generic communication systems point of view as expressed in equation (3.16). The results presented in figure 4*f* show how the signal is more attenuated as we remove each myelin sheath. It not only shows consistency between our results and validates our hypothesis but also supports findings on failing pre-synaptic AP due to demyelinating diseases [55]. We decided to sweep through the entire range of the number of myelin sheaths instead of emphasizing the cytokine storm time-dependency because it made more sense as we wanted to identify every potential demyelination effect to the axonal pathway.

**Figure 5.**
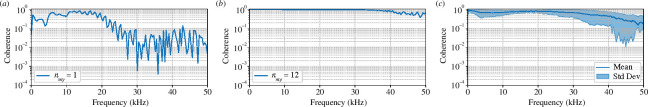
Signal coherence between $n_{my}=13$ and (*a*) $n_{my}=1$ ; (*b*) $n_{my}=12$ ; and (*c*) mean and standard deviation of the signal coherence with $1\leq n_{my}\leq 12$ . (*a*) Signal coherence with *n*_my_ = 1 (*b*) Signal coherence with *n*_my_ = 12. (*c*) Mean and standard deviation of the signal coherence for all *n*_my_.

Finally, figure 5 shows the results for the signal coherence. In figure 5*a*, we show the coherence with regard to our worst scenario, $n_{my}=1$ , in which there clearly are more oscillations in lower frequency bands when compared to figure 5*b*. Figure 5*b* shows the coherence for a light demyelination, $n_{my}=12$ , where only one sheath has been removed with regard to a healthy scenario, $n_{my}=13$ . Figure 5*c* describes the mean and standard deviation of how the coherence values ( $1\leq n_{my}\leq 12$ ) fluctuate in the 0–50 kHz spectrum. Neuronal coherence has been known to serve neuronal communication as an indicator of the efficiency of the exchange of information [58]. In this work, it is noticeable how coherence measurements show higher instability for severely demyelinated neurons in comparison with light demyelination for low- and mid-frequency ranges. As demyelination worsens, so does the reliability of the information going down the axonal channel. All coherence plots showed some fluctuations for the higher end of the frequency range. We believe the fluctuations in the coherence plots is a finite window effect. In other words, the fast Fourier transform (FFT) implicitly filters the data with a rectangular time-domain filter, which is a sinc-shaped filter in the frequency domain. For this reason, we are bound to get those lobes.

### 4.2. The linear model identification and verification

Let us observe the changes that the output of a neuron goes through when n varies, and understand these dynamics as depicted in figure 3. Reduction in the number of myelin sheaths ( n , 1≤n≤13 ) causes an increasing delay in the signal, such that spikes start later in neurons with less myelin, and they take longer to reach the peak value (in this section, we also refer to $n_{my}$ as n for improved consistency with §3.4. On the other hand, in terms of the shape of the spikes, we observe the effect on the spike height and the spike width (full width at half maximum) also in figure 3. This solution was adopted by identifying a suitable TF.

**Figure 6.**
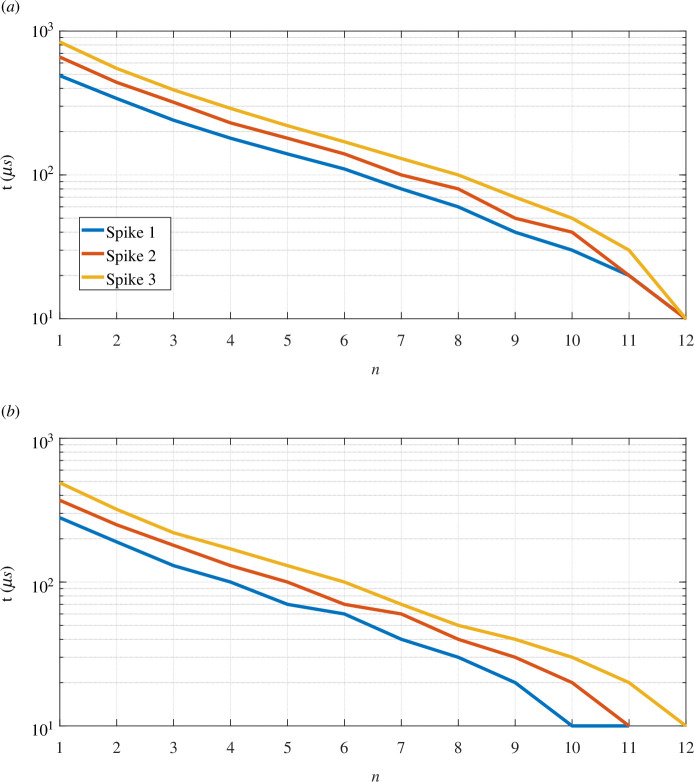
Exponential relationships between signals from different demyelinated neurons compared to the healthy n=13 case. (*a*) Time offset of spike peaks. (*b*) Time offset of spike onsets.

In figure 6*a*,*b*, we verify that time intervals represented here correspond to $\Delta t=t_{n}-t_{13}$ for 1≤n≤12 , i.e. they are the ‘lag’ observed between 13-sheath neuron, which will be the input to our model and other analysed scenarios that the model needs to approximate well, given the value of n<13 .

At this point, we can say that: (i) for 1≤n≤10 , we see an exponential decay in the ‘lag’ as n grows; and (ii) spike onsets reach the values observed in n=13 one by one ( $1^{\text{st}}$ spike for n=10 , $2^{\text{nd}}$ for n=11 , 3 ${,}^{\text{rd}}$ for n=12 ) while all the peaks reach the n=13 time values in the same case of n=12 . The first conclusion suggests that we will have a transport delay term $e^{-\tau_{n}s}$ in the TF, in which the delay $\tau_{n}$ will be an exponential function of n . The second conclusion suggests that this term cannot explain all of the dynamics: some of the lag is contributed by a real pole $-1/T_{n}$ , i.e. a term $(1+T_{n}s{)}^{-1}$ in the TF. Furthermore, after n=10 , the delay term vanishes and the only effect seen is the one of the pole (we will ignore this effect, as our approximation focus will be for the interval up to n=10 ). Again, given the linearity of the log plot, i.e. exponential nature of the curve, it is expected that $T_{n}$ is an exponential function of n .

**Figure 7.**
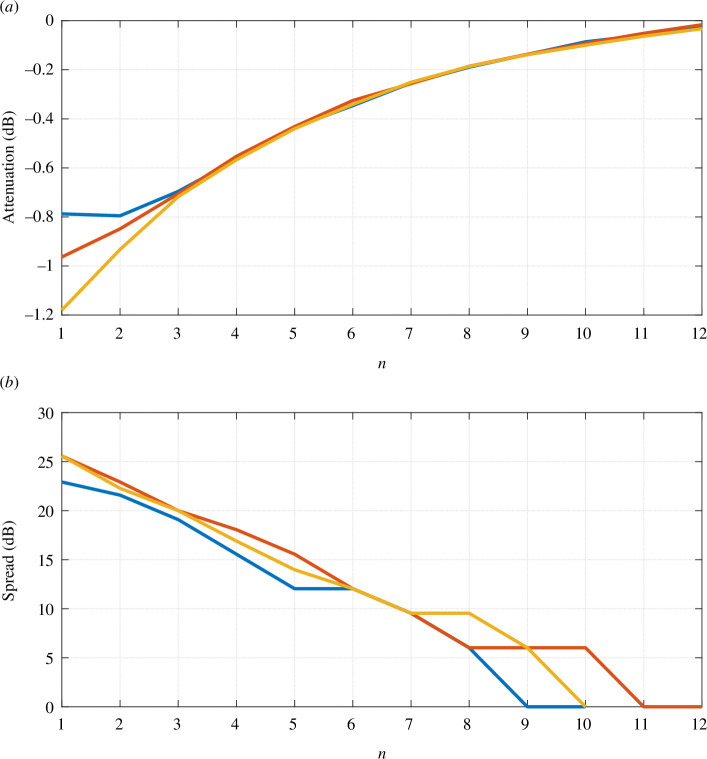
Exponential relationships between signals from different demyelinated neurons compared to the healthy n=13 case. (*a*) Ratio of spike amplitudes. (*b*) Ratio of pulse widths.

In figure7*a*,*b*, we observe the behaviour of logarithms of spike amplitudes and pulse widths in the region of interest, which suggests (i) time-invariance of the system, as all three pulses collapse in the same amplitude curve, and (ii) that the change in the pulse width requires the real pole $-1/T_{n}$ . This reasoning, graphically presented in figure 3, confirms our hypothesis about the applicability of the FOPTD TF (3.10) and its exponential coefficients from equation (3.11).

**Figure 8.**
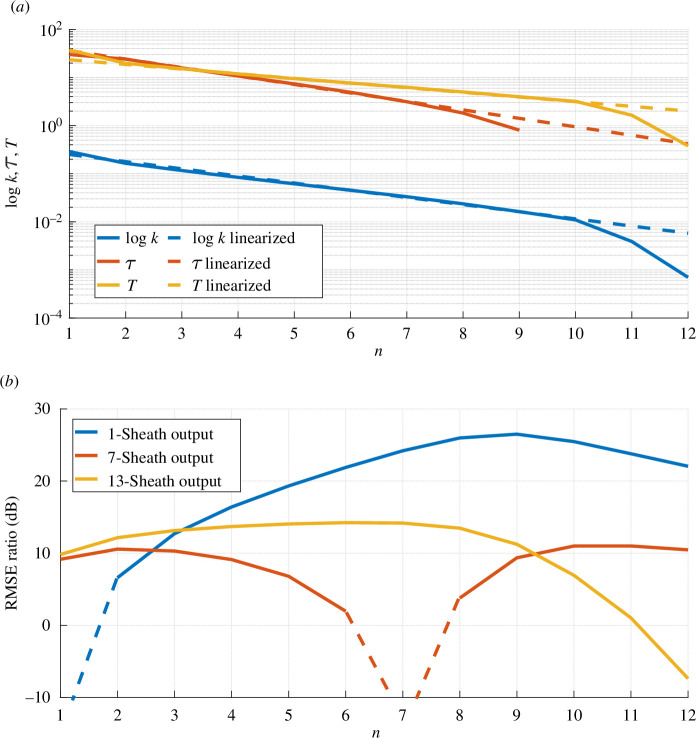
Estimation and performance for a linear model of demyelination. (*a*) Estimated parameters of the TF, and their exponential approximation. (*b*) Ratio of the RMSE for approximating a demyelinated with the single sheath case, average demyelinated neuron, or a healthy neuron and RMSE for approximating the demyelinated neurons with our TF.

The identified parameters of the model equation 3.11 are $a_{0}=0.35$ , $a_{r}=0.7$ , $\tau_{0}=54.42$ , $\tau_{r}=0.66$ , $T_{0}=20.27$ and $T_{r}=0.8$ . Those values were found with the Levenberg–Marquardt [59] numerical optimization. The iterative procedure was conducted by determining the values of $k_{n},\;\tau_{n},\;T_{n}$ for n=6 , then using those values as initial guesses for n=5 and n=7 , and subsequent values. The relationship between exponential approximation, or linearization in the *log*-domain, and the best choice of coefficients without exponential law assumption is shown in figure 8*a*.

It is expected that this would be a good approximation for the observed signals in the ‘exponential domain’, 1≤n≤10 . While the approximation can be accurate outside of this domain as well, we focus on applicability within the range, and we verified it using the RMSE metric introduced earlier in equation (3.12). For larger values of n , our numerical estimation was found τ to be zero, hence it could not be shown in a *log* plot.

Figure 8*b*, representing $M_{n}$ for 1≤n≤12 , gives the answer to the following question: if one ignores the variability in n and replaces every output signal $\left(x_{n}\right)$ with (i) one of a completely deteriorated neuron N=1 , (ii) averagely damaged neuron N=6 or (iii) a healthy neuron N=13 , how high is the amplitude of error, compared to that of our model. As expected, for n=N this ratio goes down to −∞ dB as the approximation with exact signals is perfect. However, for any other value, even N±1 , our approximation is superior (i.e. above 0 dB). It is important to emphasize that, as observed in figures 4–8, this demyelinating behaviour can be caused by other sources of degeneration. However, there are no claims those results are caused *only* by virus infections, rather we can say that it is clear from the literature that there is a specific demyelination caused by viruses, and we proposed a phenomenological model that takes into account the potential effects indirectly caused by a viral infection into the nervous system.

## 5. Conclusion

In this work, we propose an *in silico* model to quantify the impact of infection on APs. This model describes the dynamics of a cytokine storm and its relation to the extent of demyelination in a neuron. From evidence found in the literature, our phenomenological model is capable of mimicking cytokine-storm-induced demyelination’s degenerative effects. We also proposed a TF aiming towards a linear model of the demyelination process. Using traditional control and systems theory, we propose a FOPTD function that could help the design of chemical control loops for the reinforcement of myelin. It is important to emphasize that, even though we believe the linear approach described in this work can be a stepping stone for more complex systems, the whole system itself is highly nonlinear. Thus, this should be taken into account as it can affect the accuracy of future synthetically engineered therapeutics.

Although the discovery and design of new drugs requires multidisciplinary teams working together, it is important to not only understand what computational tools can offer to improve the accuracy of proposed approaches but also to reproduce cells and molecules interactions as an area of computer-aided drug development. We believe that this model can help biotechnologists as well as pharmacologists to design drugs that will be able to minimize and, hopefully, neutralize the impact of cytokine storms on myelin sheaths. This could be achieved by adding new modules to the model to facilitate the remyelination of axons, and even though the process of remyelination rarely regenerates the myelin sheaths back to their original state [60], this approach could help determine the treatment strategy needed to remyelinate the neuron, aiming to restore it as closely as possible to a healthy, fully myelinated state.

Our results show that demyelination induced by a cytokine storm not only degrades the signal but also impairs its propagation within the axon. This whole analysis led to the development of a TF that fundamentally represents the process of demyelination itself. It not only decreases the level of complexity of the system linking itself with the behaviour of an RC circuit, but also underlies the physical rationale of the system by applying biophysically plausible TF models. We believe that the proposed models will contribute to bioengineering approaches for neurodegeneration, especially demyelinating disease.

For future work, we plan to validate our computational modelling with wet-lab experiments to assess and improve the model’s accuracy. We are interested in analysing and modelling additional experiments, such as whether cytokine diffusion behaves differently in various parts of the brain due to differing diffusion coefficients. This could lead to models that incorporate multimodal data from imaging, genetic or molecular analysis, and enhanced longitudinal data. Also, different viral infections can trigger cytokine storms with different concentration levels. We expect that our proposed technique could lead to more sophisticated and precise approaches for treating neurodegeneration through computational assessment of brain damage from infection.

## Data Availability

This article has no additional data.
